# Pediatric emergency triage systems

**DOI:** 10.1590/1984-0462/2023/41/2021038

**Published:** 2022-07-15

**Authors:** Hany Simon, Claudio Schvartsman, Graziela de Almeida Sukys, Sylvia Costa Lima Farhat

**Affiliations:** a Universidade de São Paulo, São Paulo, SP, Brazil.; b Hospital Israelita Albert Einstein, São Paulo, SP, Brazil.

**Keywords:** Triage, Emergency medicine, Validation, Reliability, Triagem, Medicina de emergência, Validação, Confiabilidade

## Abstract

**Objective:**

The aim of this study was to perform a narrative review of the leading pediatric triage systems in emergency departments (EDs).

**Data source:**

Articles published between 1999 and 2019 were identified by searching the MEDLINE, EMBASE, and PubMed databases using the keywords “pediatric triage”, “pediatric assessment tools”, and “emergency department triage” with an emphasis on studies that evaluated the validation and reliability of triage systems.

**Data synthesis:**

A total of 105 articles on pediatric emergency triage systems in 12 countries were evaluated. Triage systems were divided into two groups: color-stratified triage systems and alert systems. The color-stratified triage systems included in this review were the Canadian Triage and Acuity Scale (CTAS), Manchester Triage System (MTS), Emergency Severity Index (ESI), and Australasian Triage Scale (ATS), and the alert systems included were the Paediatric Observation Priority Score (POPS), Pediatric Early Warning Score (PEWS), and Pediatric Approach Triangle (PAT). Evidence corroborates the validity and reliability of MTS, PaedCTAS, ESI version 4, PEWS, POPS, and PAT in pediatric emergency services.

**Conclusions:**

These are fundamental tools for risk classification of patients seeking treatment in EDs. Not all triage systems have been assessed for validity and reliability; nor are they well suited for all regions of the world. Employing triage systems in Brazil requires cultural adaptation and rigorous training of the local health staff, in addition to validation and reliability studies in our country, since the social and cultural context of this country differs from those where these tools were developed.

## RESUMO

**Objetivo:**

Realizar uma revisão narrativa dos principais sistemas de triagem pediátrica nos serviços de emergência.

**Fontes de dados:**

Artigos publicados entre 1999 e 2019, obtidos por meio de busca nos bancos de dados Medical Literature Analysis and Retrieval System Online (Medline), Embase e PubMed usando as palavras-chave “*pediatric triage*”, “*pediatric assessment tools*” e “*emergency department triage*”, com ênfase em estudos que avaliaram a validação e a confiabilidade desses sistemas.

**Síntese dos dados:**

Foram avaliados 105 artigos sobre sistemas de triagem na emergência pediátrica de 12 países. Os sistemas de triagem foram divididos em dois grupos: estratificados por cores e de alerta. Os sistemas de triagem com estratificação por cores incluídos nesta revisão foram: *Canadian Triage and Acuity Scale* (CTAS), *Manchester Triage System* (MTS), *Emergency Severity Index* (ESI) e *Australian Triage Scale* (ATS). Os sistemas de alerta incluídos foram: *Paediatric Observation Priority Score* (POPS), *Pediatric Early Warning Score* (PEWS) e *Pediatric Approach Triangle* (PAT). Há evidências que corroboram a validade e a confiabilidade do MTS, PedCTAS, ESI v.4, PEWS, POPS e PAT em serviços de emergência de pediátricos.

**Conclusões:**

Os sistemas de triagem são ferramentas fundamentais para o atendimento de pacientes que procuram serviços de emergência. Nem todos os sistemas de triagem foram avaliados para validação e confiabilidade nem se aplicam a todas as regiões do mundo. Para a aplicação de sistemas de triagem no Brasil, devem ser feitos adaptação cultural e treinamento do estafe de saúde local, bem como estudos de validação e confiabilidade em nosso país, dado o seu contexto social e cultural diferente daquele dos países onde essas ferramentas foram desenvolvidas.

## INTRODUCTION

Many patients seek treatment at emergency departments (EDs) every day; this often leads to overcrowding and even poses a risk to patient safety. This situation occurs worldwide and results in waiting times for patients with serious diseases. It is important to prioritize the care of patients with serious clinical conditions. In such cases, delay in care, referral to the appropriate place of care, and treatment initiation may result in increased morbidity and mortality.

The main aim of triage is to ensure priority treatment for patients requiring urgent emergency care and accurately predict the type of care they need.^1,2^ After a rapid assessment of signs and symptoms, critically ill patients are referred to the ED for immediate care, while patients with more stable conditions may wait longer for care or be referred for outpatient follow-up.^1^

The use of various triage tools in different emergency services worldwide makes it difficult to compare. This diversity also increases the difficulty of comparing heterogeneous emergency services, professional staff, and the populations served by these services.

## VALIDATION OF TRIAGE SYSTEMS

Validity refers to the ability of an instrument to discriminate what it proposes. Regarding triage systems, the assigned priority level should correspond with the actual degree of urgency.

Validation of a triage system requires best criteria to assign emergency and urgency. In the absence of a gold standard for the genuine degree of urgency, surrogate markers such as mortality, ED care, intensive care unit (ICU) admission, hospitalization, use of human and financial resources, length of stay in the ED, and cost of consultation in the ED are used to assess validity.^1,3,4^ Validation also relied on the comparison of the performance of triage system with a reference standard developed by experts.^3,5^

The validity of a triage system can also be expressed by its sensitivity and specificity. Triage systems with large proportions of under-triaged patients and with low sensitivity (i.e., too many high-urgency patients who are classified as low urgency) are unsafe. However, high sensitivity combined with low specificity can result in the treatment of many patients with low urgency as high urgency.^1,5,6^ This can cause long waits for patients with high urgency. Since it is difficult for a triage system to achieve 100% sensitivity and 100% specificity, a good balance between over-triage and under-triage is fundamental.^5,7^

Reliability of a measurement instrument is assessed by its ability to replicate the result of a measurement by other individuals in similar circumstances (inter-rater agreement) or by the same individual at different moments (intra-rater agreement).^1,5^

Reliability or replicability of the results should be as high as possible, otherwise the method is not sufficiently dependable. Reliability is described using the kappa statistic, where κ=0 indicates a random result and κ=1 shows total agreement between two or more measurements.^5,8^

## PEDIATRIC TRIAGE IN EMERGENCY SERVICES

Since triage systems have primarily been developed for the adult population,^1^ it is important to develop uniform triage systems for pediatric care. An ideal pediatric triage tool would generate reliable information in the ED, regardless of differences between clinical presentation, and would serve as an indicator of quality of health service.^2,3^

A patient’s clinical urgency does not clearly define the complexity or severity of their condition. Nevertheless, triage serves as an important measure of the critical time for medical intervention.^7^

However, currently, there is no standardized tool for pediatric patients that is used routinely. One of the difficulties in establishing such a standard tool is the variation in clinical parameters in different age groups. An additional concern is that a critically ill child may initially appear stable but then rapidly deteriorate. Thus, a triage tool may not provide sufficient warning in the initial stages to ensure adequate care.^9,10^

Some pediatric alert triage systems that allow rapid recognition of the severity of the patient’s condition utilize a rapid assessment of physiological parameters; any alteration in these parameters indicates the need for prompt medical evaluation. However, recording vital signs alone is not adequate to identify critically ill patients in an ED.^8,11^

Color scales can have three (traffic light system), four, or five levels. Some of these instruments are used in private institutions without sufficient documentation and despite their questionable reliability.^11,12^

Unlike instruments with three levels of classification, instruments with five levels correlate, in addition to quality assurance, with management indicators including resource utilization, hospital admission rates, length of stay in the ED, transfer to the ICU, or mortality rate. They have been recommended by national and international societies for emergency triage.^8,13^ These levels are divided in increasing numerical or color order from levels 1 to 5, corresponding to immediate, very urgent, urgent, standard, and nonurgent.^14^

## LEADING PEDIATRIC EMERGENCY TRIAGE SYSTEMS

Magalhães-Barbosa et al. recently published systematic reviews evaluating the reliability^15^ and validity^16^ of pediatric emergency triage systems.

The authors concluded that there is some evidence to support the validity of the Manchester Triage System (MTS), Paediatric Canadian Triage and Acuity Scale (PaedCTAS), and Emergency Severity Index (ESI) version 4 for the care of children in ED in their countries. However, there is a need to improve the sensitivity and reduce the patient misclassification rates.

The authors also concluded that there is some evidence supporting the reliability of these scores, but they remain limited to the countries in which they were developed. Thus, efforts are needed to improve their quality and adapt them for countries with professionals with different qualifications and sociocultural settings.^15,16^

## FIVE-LEVEL PEDIATRIC TRIAGE INSTRUMENTS

The leading five-level pediatric triage instruments are the CTAS: Table 1, MTS: Table 2, ESI: Table 3, and Australasian Triage Scale (ATS).

**Table 1:** Studies evaluating the Canadian Triage and Acuity Scale.

Author and year	Objective	Population	Study type	Conclusions
Gouin et al.^6^ 2005	Performance comparison between PaedCTAS and PRISA.	1281	Prospective	More patients were classified as high severity with PaedCTAS with a high level of acuity but with less ability to predict admissions. The ability to predict interventions was similar in both.
Gravel et al.^21^ 2013	Association between triage levels of CTAS and surrogate markers validity for real-life children triaged in ED.	550,940	Retrospective cohort	There was a strong association between triage level and multiple markers of severity that suggests validity of CTAS for children.
Gravel et al.^50^ 2007	Evaluation of inter-evaluator agreement.	29 nurses	Experimental in two phases	The computerized version showed statistically significant improvements in agreement between nurses.
Bergeron et al.^22^ 2004	Assessment of inter-evaluator agreement, between nurses and emergency pediatricians.	29 nurses 15 pediatricians	Cross-sectional	The study sent 55 scenarios to physicians and nurses. The level of agreement and accuracy was moderate between physicians and nurses and did not vary according to each group’s experience and occupation.
Ma et al.^23^ 2008	Assessment of the correlation between PaedCTAS levels and the use of resources.	1618	Prospective	PaedCTAS levels correlated well with the use of resources by pediatric ED patients.
Gravel et al.^24^ 2012	Assessment of inter-evaluator agreement and validation of the triage level and severity markers.	1464	Prospective	The study found good agreement between nurses in a pediatric ED and good association with severity markers.

PaedCTAS, Paediatric Canadian Triage and Acuity Scale; PRISA, Pediatric Risk of Admission; ED, emergency department.

**Table 2:** Studies evaluating the Manchester Triage System.

Author and year	Objective	Population	Study type	Conclusions
Roukema et al.^28^ 2006	Validation in a pediatric ED	1065	Prospective	The MTS showed moderate validity in pediatric ED.
Van Veen et al.^27^ 2008	Validation in a pediatric ED	13,554	Observational, prospective	The MTS showed moderate validity in pediatric ED. Triage was more difficult for younger patients and those with medical problems.
Van der Vulp et al.^51^ 2008	Validation and assessment of reliability of triage systems	50 scenarios	Prospective	Assessment of emergency scenarios and reassessment 19 days later. Moderate and substantial inter-evaluator reliability and high test/retest reliability were observed.
Van Veen and Moll^1^ 2009	Validation and assessment of reliability of triage systems.		Literature review	The MTS was a valid pediatric emergency triage system with good reliability.
Van Veen et al.^52^ 2010	Assessment of the repeatability of the MTS in children	20 scenarios	Prospective	The MTS showed good to very good repeatability in pediatric ED.
Mirhaghi et al.^29^ 2017	Assessment of the reliability of the MTS.		Meta-analysis	The MTS showed an acceptable level of overall reliability in a pediatric ED.
Magalhães-Barbosa et al.^15^ 2019	Assessment of the reliability of pediatric emergency triage systems		Systematic review	There was some evidence of MTS reliability, but most studies were limited to the countries where the scale was developed.

ED: emergency department; MTS: Manchester Triage System. ESI, Emergency Severity Index; ED, emergency department.

**Table 3:** Studies evaluating the Emergency Severity Index.

Author and year	Objective	Population	Study type	Conclusions
Tanabe et al.^54^ 2004	Evaluate ESI v.3 reliability and validation in ED.	403	Retrospective	Excellent level of reliability and correlation with resource utilization and length of stay in the ED.
Baumann et al.^53^ 2005	Assessment of ESI v.3 reliability and validity in children.	510 (at the validation stage)	Study in two phases: 1. Retrospective 2. Observational prospective	Excellent level of agreement between evaluators. Demonstrated validity in the task of triage and resource utilization.
Van Veen and Moll^1^ 2009	Validation and reliability of the ESI v.3 triage system.		Literature review	ESI v.3 was a valid pediatric emergency triage system with good reliability.
Durani et al.^30^ 2009	To measure ESI v.4 reliability and agreement between evaluators and between physicians and nurses.	20 scenarios	Prospective	The ESI v.4 showed high reliability and agreement (83%) between emergency personnel and nurses.
Travers et al.^31^ 2009	Evaluation of reliability of ESI v.4 and its validation in pediatric EDs for all age groups.	40 scenarios 1671 patients	Prospective	Moderate reliability. The study identified scenarios in which nurses had difficulty in performing triage consistently.
Green et al.^32^ 2012	Assessment of ESI v.4 reliability and validation in pediatric ED.	780	Study in two phases: 1. Retrospective 2. Prospective cohort	High agreement between nurses and physicians. ESI v.4 predicted hospital admission, length of stay, and use of resources in pediatric ED.
Jafari-Rouhi et al.^33^ 2013	Assessment of ESI v.4 reliability and validation in pediatric ED.	20 scenarios 1104 patients	Study in two phases: 1. Retrospective 2. Prospective cohort	There was agreement between nurses and physicians and reliability when performed by experienced professionals.
Magalhães-Barbosa et al.^15^ 2019	Assessment of the reliability of pediatric ED triage systems		Systematic review	There is some evidence supporting the reliability of ESI v.4, but most studies were limited to the countries where the scales were developed.

### Canadian Triage and Acuity Scale

In 1997, the Canadian Association of Emergency Physicians (CAEP) in conjunction with the National Emergency Nurses Association (NENA) developed the five-level CTAS for ED;^17^ the application of this system is mandatory in most Canadian provinces. In 2001, the CAEP in collaboration with NENA and the Canadian Paediatric Society proposed the PaedCTAS, a five-level triage scale for pediatric patients that was derived from the adult scale.^18^

The PaedCTAS has a physiological approach (i.e., assessment of appearance, neurological signs, respiratory rate, heart rate, and perfusion) and uses complex symptoms to assign triage levels. The instrument, like the adult version, is composed of five triage levels, each consistent with the degree of severity of the clinical presentation and time for medical evaluation and intervention. Specific criteria were created to assign patients to different levels of emergency care. Therefore, patients at level 1 (blue-resuscitation) should be seen immediately, patients at level 2 (red-emergent) should be seen in 15 min, and patients at levels 3 (yellow-urgent), 4 (green-less urgent), and 5 (white-nonurgent) should receive medical care within 30 min, 1 h, and 2 h, respectively.^19^

Gouin et al.^3^ compared the performance of the PaedCTAS to that of a previously used triage tool in a tertiary pediatric ED using the percentage of admissions, diagnostic and therapeutic interventions, and Pediatric Risk of Admission (PRISA) score.^20^ The ability to predict admission was higher in the PRISA. The ability to predict interventions such as blood culture collection and intravenous fluid boluses was similar between the triage tools.

In their literature review, van Veen et al.^1^ evaluated the reliability and validation of triage systems in pediatric ED and concluded that the PaedCTAS is a valid system for pediatric triage in EDs, with moderate reliability.

Gravel et al. in a retrospective cohort evaluated the association between triage assessment levels using the CTAS and outcomes in children presenting to 12 pediatric EDs during a 1-year period. There was a strong association between triage level and admission to the ICU, probability of leaving without medical consultation, and length of stay, suggesting validity of the CTAS for children.^21^

Bergeron et al. assessed the agreement between evaluators and concluded that PaedCTAS has a weak to moderate level of agreement. The 95% confidence interval (95%CI) was low among pediatric emergency physicians (0.39 [0.38–0.41]) and moderate for nurses (0.51 [0.50–0.52]).^22^

Ma et al. assessed the association of PaedCTAS level and resource utilization in patients in a pediatric ED and found the average costs for laboratory, microbiology, imaging, and total clinical investigations increased with increasing acuity.^23^

Gravel et al. evaluated PaedCTAS in a prospective study of nine pediatric EDs. The inter-rater agreement between triage and research nurses and the association between triage level and severity markers were evaluated. The level of agreement between observers showed a good kappa index of 0.74 (95%CI 0.71–0.76). The rate of hospitalization was 30, 8.3, 2.3, and 2.2% for triage at levels 2, 3, 4, and 5, respectively. There was also a strong association between the triage level and the need for hospital resources and number of days of hospitalization, that is, more severe triage levels required a higher degree of intervention.^24^

### Manchester Triage System

The MTS contains 52 flowcharts for different problems, including specific flowcharts for pediatric patients. The flowcharts contain general and specific discriminators depending on patient signs and symptoms.^25^

Discriminator selection defines the level of urgency. Patients at level 1 (red) must be seen immediately by a physician. At level 2 (orange-very urgent), medical care must be provided within 10 min. At levels 3 (yellow-urgent), 4 (green-standard), and 5 (blue-nonurgent), patients must be seen within 60 min, 2 h, and 4 h, respectively.^14,25,26^

A major validation study of the MTS was conducted using a computerized triage system that calculated the flowchart and urgency levels for the problems presented and compared them to a standard five-level reference triage scale. Agreement in urgency level between the two triage systems was observed in 34% of patients. The MTS resulted in over-triage and under-triage estimation in 54 and 12% of patients, respectively. The disagreement was greater for younger children and patients with medical problems.^27^

Roukema et al. evaluated the validity of the MTS using information on vital signs, resources used, and hospitalization. The sensitivity and specificity of the MTS to detect emergency cases were 63 and 78%, respectively. Under-triage occurred in 15% of patients and over-triage occurred in 40% of patients.^28^

Recent reviews by Mirhagui et al. in 2017 (a meta-analysis) and Magalhaes-Barbosa et al. in 2019 (a systematic literature review) showed acceptable reliability of the MTS; however, further studies are needed.^15,29^

### Emergency Severity Index

The ESI is a five-level system developed in the United States. Levels 1 and 5 correspond to the highest and lowest severities, respectively. Patients requiring immediate care and at high risk are assigned to level 1 and should be seen immediately.^12^

The fourth version of the ESI included a specific flowchart for febrile children that considers their age, temperature, and immunization status.^12^

On assessing the reliability of the ESI version 4, Durani et al.^30^ found an 83% agreement rate between physician and nurse responses to pediatric scenarios. They considered it a reliable tool when used by experienced professionals.

The first large-scale study on the reliability and validity of the ESI version 4 for pediatric triage was conducted by Travers et al.^31^ The interobserver reliability was assessed through pediatric scenarios and double triage, and validation was done through an outcome assessment after the ESI stratification, which included hospital admission. The authors concluded that the inter-rater reliability was moderate, finding inconsistencies in triage was more in acute patients, those under 1 year of age, and those with preexisting health conditions (except for trauma).

Green et al.^32^ evaluated the reliability and validation of ESI version 4. The tool was validated through a retrospective study of 780 pediatric patients in an ED. The authors concluded that the instrument was reliable for pediatric ED triage with a high degree of agreement between nurses and physicians. It was also a valid predictor of hospital admission, length of stay in ED, and use of resources.

Jafari-Rouhi et al.^33^ evaluated the reliability of the ESI version 4 and concluded that it was a reliable instrument in experienced hands. The authors concluded that the instrument was useful in challenging triage cases.

### Australasian Triage Scale

The ATS is used in public hospitals in Australia to ensure that patients receive timely care according to their clinical urgency.

All patients are assigned a triage score by a trained nurse or physician upon their arrival at the ED. The scale categories range from level 1 (most urgent, requiring immediate care and treatment) to level 5 (least urgent).^34^ The same triage standards are used for adults, adolescents, and children.

A limited number of studies have assessed the applicability of the ATS in the pediatric population. These studies reported poor to moderate reliability.^35^

In their meta-analysis, Ebrahim et al.^36^ evaluated the reliability of the ATS in six studies, only one of which was pediatric. They concluded that the ATS showed an acceptable level of reliability.

### Alert triage systems

The main instruments of pediatric triage for alert and rapid recognition of severity are the Paediatric Observation Priority Score (POPS), Pediatric Early Warning Score (PEWS), and Pediatric Assessment Triangle (PAT) (Table 4). These classification systems are based on the rapid assessment of physiological parameters that, if altered, indicate a need for prompt medical evaluation.

**Table 4:** Studies evaluating the Alert triage systems.

Author, year, and scale	Objective	Population	Study type	Conclusions
Roland et al.^37^ 2016 – POPS	Validation of the POPS as a triage tool.	936	Observational prospective	The POPS demonstrated an ability to help healthcare professionals make decisions.
Cotterill et al.^38^ 2016 – POPS	Investigation of the ManchEWS and POPS ability to predict hospital admission.	2068	Prospective cohort	The POPS was a more accurate predictor of risk for hospital admission in EDs than the ManchEWS. The POPS appeared to be clinically appropriate for use in pediatric ED.
Roland et al.^39^ 2017 – POPS	To examine the utility of the POPS in a pediatric ED and determine its performance.	3323	Prospective cohort	It is feasible to implement the POPS in EDs; it had performance characteristics similar to those of the original development site.
Riaz et al.^40^ 2018 – POPS	To refine and test the diagnostic accuracy of the PAT-POPS in predicting admission and discharge of children in ED.	16,000 Minimum estimated sample	Observational retrospective	This predictive tool can help in hospitalization and discharge decisions for children and youth in ED.
Bradman et al.^42^ 2008 – PEWS	To determine whether the PEWS can identify children requiring hospitalization during triage	424	Observational prospective	The PEWS showed low sensitivity in predicting the need for hospitalization
Seiger et al.^43^ 2013 – PEWS	To compare PEWS performance in EDs to predict hospital or ICU stays	17,943	Prospective cohort	The discriminatory ability of the PEWS was moderate to good for predicting ICU admission and poor to moderate for predicting hospital admission.
Gold et al.^44^ 2014 – PEWS	To determine whether the PEWS predicts the need for ICU admission	12,306	Observational prospective	High scores were associated with the need for ICU admission. The study did not demonstrate that the PEWS could determine hospital discharge or predict deterioration.
Horeczko et al.^46^ 2013 – PAT	To determine the reliability and accuracy of the PAT as applied by nurses.	528	Observational prospective	The PAT quickly and reliably identified acute pediatric patients and their pathophysiological conditions.
Fernandez et al.^47^ 2017 – PAT	To evaluate the association between PAT findings in triage and severity markers of patients in pediatric ED.	302,103	Retrospective cohort	PAT findings were an independent risk factor for hospital admission, ICU admission, and length of ED stay. It is a valid tool to identify more severe patients as a first step in the triage process.
Paniagua et al.^48^ 2017 – PAT	To assess the performance of the PAT in predicting hospitalization, ICU admission, and length of stay at the ED in children with asthma exacerbation.	14,953	Retrospective cohort	PAT is a good predictor of hospital admission. Abnormality in PAT is an independent risk factor for hospital admission and length of stay.

POPS: Paediatric Observation Priority Score; ManchEWS: Manchester Children’s Early Warning System; PRISA: Pediatric Risk of Admission; PEWS: Pediatric Early Warning Score; PAT: Pediatric Approach Triangle; ED: emergency department; ICU: intensive care unit.

Alert systems are good tools to identify patients in need of immediate care and recognize the clinical deterioration of patients under observation or inpatients. However, these systems do not stratify patients according to different levels of severity or priority of care required and cannot predict the need for logistical, financial, or skilled-labor support.

### Paediatric Observation Priority Score

The POPS is a risk stratification scale developed in the United Kingdom to assess children in ED. It aims to identify patients with severe health impairment and facilitate hospital discharge of patients with mild symptoms.

The POPS incorporates both objective physiological parameters, such as heart rate and temperature, and subjective observational parameters, such as the level of concern. It also considers any chronic conditions of the patient. Each parameter of the POPS ranges from 0 to 2, and patients with scores >8 should be transferred to the ED.^37^

Cotterill et al.^38^ collected prospective data from over 2,000 patients under the age of 16 and compared the POPS and Manchester Children’s Early Warning Score’s (ManchEWS) ability to predict hospital admission in a pediatric ED.

Roland et al.^39^ evaluated the basic characteristics of the POPS in four pediatric EDs and in patients under 16 years of age according to three outcomes: discharge from ED, discharge from ED with a return visit within 1 week, and admission for more than 24 h. The implementation of the POPS was feasible and had similar performance characteristics in predicting admissions and safety at hospital discharge, services that were outside its original

Riaz et al.^40^ conducted an observational study in three hospitals for 12 months in 16,000 patients to assess the accuracy of the POPS, using hospital admission and safe discharge as predictors. The authors concluded that the POPS was an easy tool to implement, and it did not require additional infrastructure.

### Pediatric Early Warning Score

The PEWS considers patient behavior along with cardiovascular and respiratory status parameters, each given a score between 0 and 3, with additional points for persistent vomiting and the need for continuous nebulization. The PEWS is not age-specific and provides a 13-point scale of clinical severity that is used to identify patients at risk of clinical deterioration and in need of intensive care.^41^

Bradman et al.^42^ assessed whether the PEWS could accurately identify children, aged 0–16 years, at risk for admission or requiring discharge in ED. The authors concluded that the PEWS had limited value in predicting hospital admission.

Seiger et al.^43^ evaluated children under 16 years of age in an ED in the Netherlands for 3 years using the PEWS. The area under the receiver operating characteristic (ROC) curve predicting ICU stay was moderate to good and that predicting admission was poor to moderate. The sensitivity and specificity varied widely.

Gold et al.^44^ assessed the outcomes of 12,306 patients under 21 years of age who were evaluated by nurses using the PEWS; they reported excellent inter-evaluator reliability (coefficient: 0.91). The study showed that high scores in the ED were associated with the need for ICU admission. However, as a single tool, it did not have sufficient ability to determine hospital discharge or predict deterioration.

### Pediatric Assessment Triangle

In 2000, the American Academy of Pediatrics introduced a new rapid access tool, the PAT, which allowed physicians to assess the general presentation of sick children, establish the severity of their clinical presentations, and determine the type of emergency intervention required.

The PAT is a rapid assessment tool that can be performed in 30 to 60 s and does not require equipment, only visual and auditory evaluations. The three components of the PAT are appearance, respiratory function, and skin circulation. Together, these components reflect the general states of oxygenation, ventilation, perfusion, and brain function of the child.^10,45^

Horeczko et al.^46^ evaluated the accuracy of the PAT applied by nurses in a pediatric ED. The assessment using this score quickly and reliably identified the medical conditions of pediatric patients based on the following pathophysiological categories: respiratory distress, respiratory failure, shock, cardiopulmonary failure, and central nervous system and/or metabolic disorders.

In their retrospective study of more than 300,000 cases, Fernandez et al.^47^ assessed the associations between PAT findings during triage and severity markers in pediatric ED. The authors concluded that abnormal findings in the PAT, when it was used by trained nurses, were associated with a high probability of hospitalization and ICU admission as well as with long stay durations in the ED.

Paniagua et al.^48^ evaluated the performance of the PAT score, triage levels, pulmonary score, and initial oxygen saturation in predicting hospitalization for acute exacerbation of asthma in children. They showed that the PAT was useful for identifying the most severe patients and those in need of hospitalization after the initial treatment. The PAT components most associated with hospitalization were labored breathing and skin circulation.

## DISCUSSION

The ED is the hospital’s main point of entry for most patients. The volume of care in the ED cannot be predicted, and most patients who come to the ED will not be admitted and treated immediately and simultaneously. Serious illnesses, or life-threatening conditions, need to be identified quickly, minutes after their arrival, and that requires an efficient triage system. Pediatric triage systems must be structured and implemented in all emergency services to ensure that patients with severe conditions can be identified immediately and quickly directed to the definitive treatment site.

Triage in a pediatric emergency service is a task that is both challenging and complex. Children bring some challenges to the triage systems, since they may exhibit a variation of vital signs depending on their age group and may also present nonspecific clinical symptoms, besides requiring the assistance of an interlocutor during care.^49^

The implementation of a pediatric triage system in the ED is divided into two very imperative phases: planning and transition. Both phases require the engagement of all involved parties, including administrative, information technology, medical, and multiprofessional staff. In determining the best triage system for each service, we must first consider the technological resources available, the ease of in-site implementation, and the quality of the assessment tool. This choice must be made in conjunction with the attending medical team and the administrative area.

The triage tool, in addition to cataloging patients within emergency care, must also be able to estimate the resources each will require from each sector. Differences in human resources, technology, professional qualification, and health policies may interfere with its performance. Realistically, the implementation of a pediatric triage system in an ED may take between 9 and 12 months.^8^

Many pediatric ED triage systems were validated and deemed reliable in the countries where they were developed, or in countries with similar sociodemographic and cultural conditions. There is evidence to support the validity and reliability of tools such as the MTS, PaedCTAS, ESI version 4, PEWS, POPS, and PAT in pediatric emergency services.

Brazil is a country of continental dimensions and with distinct and heterogeneous regions with various geographical, cultural, economic, and health systems. Currently, we lack the exact number of pediatric emergency services in the country. To implement the triage systems in Brazil, we must adapt it to the local culture and offer rigorous training to the local health staff. In addition, validation and reliability studies must be carried out, since the social and cultural context of this country differs from those where such tools were developed.

It is also necessary that the government and the regional pediatric societies to catalogue and computerize the various pediatric emergency services throughout the country. In this context, the inclusion of a pediatric triage system is essential for improving medical care and better manage the available resources.

Triage systems were developed to rank patients for treatment in situations where there is a shortage of resources and high demand. They are fundamental tools for risk classification of patients seeking treatment in ED and ensure that those with severe conditions receive priority care. Pediatric emergency triage systems used worldwide can be divided into risk stratification and rapid–response systems. Not all triage systems have undergone validity and reliability assessments; nor are they well suited for all regions of the world.

In Brazil, not all emergency health care service which take care of children and adolescent are computerized or have triage systems to help in prioritizing patients with the most urgent management need. Due to the great territorial extension and marked regional differences, it is a great challenge to determine the ideal pediatric emergency triage system. Expanding the knowledge of pediatricians about the existing ones will encourage EDs to implement a pediatric emergency triage system to improve the quality of care in this area.
